# Prevention of the β-amyloid peptide-induced inflammatory process by inhibition of double-stranded RNA-dependent protein kinase in primary murine mixed co-cultures

**DOI:** 10.1186/1742-2094-8-72

**Published:** 2011-06-23

**Authors:** J Couturier, M Paccalin, M Morel, F Terro, S Milin, R Pontcharraud, B Fauconneau, G Page

**Affiliations:** 1 Research Group on Brain Aging, GReViC EA 3808, 6 rue de la Milétrie BP 199, 86034 Poitiers Cedex, France; 2 Department of Geriatrics, Unity of ultrastructural and experimental Pathology Poitiers University Hospital, 2 rue de la Milétrie BP 577, 86021 Poitiers Cedex, France; 3 CIC, INSERM 0802, Unity of ultrastructural and experimental Pathology Poitiers University Hospital, 2 rue de la Milétrie BP 577, 86021 Poitiers Cedex, France; 4 Group of cellular neurobiology: cell homeostasis and pathologies, EA 3842, 2, rue du Dr Raymond Marcland, 87025 Limoges CEDEX, France; 5 Laboratory of histology, cellular biology and cytogenetic, Hospital "Mother and children"8 avenue D. Larrey, 87042, Limoges CEDEX, France; 6 Department of Anatomy and Pathologic Cytology, Unity of ultrastructural and experimental Pathology Poitiers University Hospital, 2 rue de la Milétrie BP 577, 86021 Poitiers Cedex, France

## Abstract

**Background:**

Inflammation may be involved in the pathogenesis of Alzheimer's disease (AD). There has been little success with anti-inflammatory drugs in AD, while the promise of anti-inflammatory treatment is more evident in experimental models. A new anti-inflammatory strategy requires a better understanding of molecular mechanisms. Among the plethora of signaling pathways activated by β-amyloid (Aβ) peptides, the nuclear factor-kappa B (NF-κB) pathway could be an interesting target. In virus-infected cells, double-stranded RNA-dependent protein kinase (PKR) controls the NF-κB signaling pathway. It is well-known that PKR is activated in AD. This led us to study the effect of a specific inhibitor of PKR on the Aβ42-induced inflammatory response in primary mixed murine co-cultures, allowing interactions between neurons, astrocytes and microglia.

**Methods:**

Primary mixed murine co-cultures were prepared in three steps: a primary culture of astrocytes and microglia for 14 days, then a primary culture of neurons and astrocytes which were cultured with microglia purified from the first culture. Before exposure to Aβ neurotoxicity (72 h), co-cultures were treated with compound C16, a specific inhibitor of PKR. Levels of tumor necrosis factor-α (TNFα), interleukin (IL)-1β, and IL-6 were assessed by ELISA. Levels of P_T451_-PKR and activation of IκB, NF-κB and caspase-3 were assessed by western blotting. Apoptosis was also followed using annexin V-FITC immunostaining kit. Subcellular distribution of P_T451_-PKR was assessed by confocal immunofluorescence and morphological structure of cells by scanning electron microscopy. Data were analysed using one-way ANOVA followed by a Newman-Keuls' post hoc test

**Results:**

In these co-cultures, PKR inhibition prevented Aβ42-induced activation of IκB and NF-κB, strongly decreased production and release of tumor necrosis factor (TNFα) and interleukin (IL)-1β, and limited apoptosis.

**Conclusion:**

In spite of the complexity of the innate immune response, PKR inhibition could be an interesting anti-inflammatory strategy in AD.

## Background

One hundred years ago, Fisher [1] proposed that the deposition of a foreign substance in the human cortex of patients with Alzheimer's disease (AD), later identified as fibrillated amyloid-β peptide (Aβ), could induce a local inflammatory reaction associated with regenerative changes in the surrounding neurons. The innate immune response in AD is marked by the production of various complement components (C1q, C3, C5) and formation of the terminal membrane attack complex, resulting in attraction and activation of microglia and astrocytes [2-6]. Both microglia and astrocytes produce multiple pro-inflammatory factors, including cytokines (tumor necrosis factor-α (TNFα), interleukin (IL)-1, and IL-6), chemokines (CC- and CXC-chemokine ligands such as CCL2, 3, and 5; and CXCL10), reactive oxygen species, and cyclooxygenase 2 (COX2); and express various complement receptors [7,8]. This inflammatory response aims to enhance the clearance of Aβ by the phagocytic role of both microglia and astrocytes. Although activation of the complement system or a lipopolysaccharide (LPS) treatment in amyloid precursor protein (APP) transgenic mice increases phagocytosis of Aβ and might limit pathology by activating immune responses [8,9], the beneficial role of inflammation in AD does not seem to be sufficient to halt or reverse the disease. It fails to slow progression of the major histopathological hallmarks (amyloid plaques and neurofibrillar tangles) and cognitive impairment. The innate immunity system might be neuroprotective as far as phagocytosis is elicited, but later in the disease proinflammatory responses could turn the innate immunity into the driving force in AD pathogenesis.

Increasing evidence suggests that inflammation significantly contributes to the pathogenesis of AD. It is known that Aβ oligomers and fibrils, as danger-associated molecular patterns (DAMPs), can interact with different pattern recognition receptors (PRRs) such as scavenger receptors, toll-like receptors (TLRs), and the receptor for advanced glycation end products (RAGE) in both glial cells and neurons [10,11]. PRRs can trigger phagocytic uptake of Aβ but also can induce proinflammatory signaling pathways such as IκB kinase (IKK), Jun kinase (JNK) p38 and glycogen synthase kinase 3β (GSK-3β) [10].

Many cytokines such as TNFα and IL-1β, and chemokine signaling (CXCR2 signaling) can promote Aβ production by modulating γ-secretase activity in neurons [12,13]. Some studies have also demonstrated that IL-1β induces phosphorylation of tau protein and triggers formation of paired-helical filaments (PHFs) which aggregate into neurofibrillary tangles [14,15]. Inflammation in AD could also trigger functional impairment since inflammatory molecules such as TNFα, IL-1 and IL-6 are able to suppress hippocampal long term potentiation [16,17]. Furthermore, many studies have shown a significant increase of various inflammatory mediators in plasma and in peripheral blood mononuclear cells (PBMCs) of patients with AD compared to age-matched controls [18,19].

In addition, many prospective epidemiological studies have indicated that non-steroidal anti-inflammatory drugs (NSAIDS) might delay the onset and the progression of AD [20]. However, clinical trials with COX-2 inhibitors have yielded negative results, and the relevance of specific COX inhibitors and other NSAIDS has become more and more questionable [21]. There are many reasons to explain the failure of these trials: timing of treatment, dosing, and the specificities of administrated NSAIDS are the most frequently cited. A recent small, open-label pilot study suggested that inhibition of the inflammatory cytokine TNF-α with perispinal administration of etanercept, a potent anti-TNF fusion protein, might lead to sustained cognitive improvement in patients with mild, moderate, or severe AD [22]. These results need to be confirmed.

The cellular and molecular components of the innate inflammatory response associated with slowly progressive degenerative disease are not clearly identified. In this response, Aβ could involve different PRRs, activating protein kinases such as IKKs which trigger proinflammatory responses *via *nuclear factor-kappa B (NF-κB), known as the major transcriptional factor of a wide range of cytokines, that could in turn maintain NF-κB activation and establish a positive autoregulatory loop that could amplify the inflammatory response and increase the duration of chronic inflammation [23]. The modulation of NF-κB activation in AD may be a neuroprotective strategy. A recent study revealed that an inhibitor of NF-κB ameliorates astrogliosis but has no effect on amyloid burden in APPswePS1dE9 [24], probably due to late timing of the treatment after the beginning of amyloid deposits. The IKK/NF-κB signaling pathway is under the control of other kinases, in particular the double-stranded RNA-dependent protein kinase (PKR), well described in AD and associated with degenerating neurons and cognitive decline [12,25-30]. Indeed, in studies using different virus-infected cells, it has been shown that PKR can phosphorylate IKK, which phosphorylates IκB, leading to disruption of the cytosolic IκB-NF-κB complex. This allows NF-κB to translocate from the cytoplasm to the nucleus, where it binds to its specific sequences of DNA called response elements of the target genes, including those involved in the immune response (IL-2), inflammatory response (TNFα, IL-1, IL-6), cell adhesion (I-CAM, V-CAM, E-selectin) cell growth (p53, Ras, and c-Myc) and apoptosis (TNF receptor-associated factor 1 and 2) [31-33]. Furthermore, it has been shown that TNF-induced NF-κB activation, IKK activation, IκBα phosphorylation, IκBα degradation and NF-κB reporter gene transcription are all suppressed in PKR gene-deleted fibroblasts, underlining the fact that NF-κB is a downstream target of PKR [34].

The aim of the present study was to determine whether PKR can control activation of the NF-κB pathway and cytokine production (TNF, IL-1β, and IL-6) in primary mouse co-cultures that contain the three main cellular actors in brain: neurons, astrocytes and microglia. While neurons are traditionally passive bystanders in neuroinflammation, they are able to produce inflammatory mediators such as IL-1β, IL-6, TNFα [15,35,36]. Although this integrated *in vitro *model does not correspond exactly to the brain environment, it includes the major cell types of brain and maintains the interactions between these three cellular actors which could modulate the inflammatory response of each one.

For this purpose, before exposure to Aβ neurotoxicity, co-cultures were treated with compound C16, a specific inhibitor of PKR [37]. Analysis of results shows that inhibition of PKR prevents activation of NF-κB, associated with a strong decrease in production and release of TNFα and IL-1β, and limited apoptosis. Keeping in mind the complexity of the innate immune response, inhibition of PKR could be an interesting strategy to rescue the inflammatory process in AD.

## Methods

### Chemical products

Sodium fluoride (NaF), phenylmethylsulfonyl fluoride (PMSF), protease and phosphatase inhibitor cocktails, dithiothreitol (DTT), 0.01% poly-L-lysine solution, Percoll^®^, sterile filtered dimethylsulfoxide Hybri-Max^® ^(DMSO), Triton X-100, paraformaldehyde (PFA), annexinV-fluorescein isothiocyanate (FITC) apoptosis detection kit and all reagent-grade chemicals for buffers were purchased from Sigma (St Quentin Fallavier, France); DMEM (1 g/L), MEM and Neurobasal media, B-27 Supplement, 200 mM L-glutamine, 5,000 units of penicillin (base) and 5,000 μg of streptomycin (base)/mL (PS) mixture, 0.5 g/L Trypsin/0.2 g/L EDTA 4Na, Fetal Bovine Serum, Certified (FBS), Horse Serum, NuPAGE^® ^Novex^® ^Bis-Tris Mini Gels, NuPAGE^® ^LDS 4X LDS Sample Buffer, NuPAGE^® ^Sample Reducing Agent (10X), NuPAGE^® ^MES SDS Running Buffer and NuPAGE^® ^Antioxidant, iBlot^® ^Gel Transfer Device (EU), the Prolong Gold antifade reagent with 4',6-diamidino-2-phenylindole (DAPI) and the Zenon mouse IgG labelling kit from Gibco-Invitrogen (Fisher Bioblock Scientific distributor, Illkirch, France); the imidazolo-oxindole compound C16 from Merck Chemicals Calbiochem^® ^(Nottingham, UK). For western blot, primary antibodies and secondary anti-rabbit IgG antibody conjugated with horseradish peroxydase were purchased from Cell Signalling (Ozyme, St Quentin Yvelines, France) excepted anti-P_T451_-PKR from Eurogentec (Seraing, Belgium), anti-β tubulin and anti-β actin from Sigma (St Quentin Fallavier, France), anti-amyloid peptide (clone WO2, recognizes amino acids residues 4-10 of Aβ) from Millipore (St Quentin-Yvelines, France), peroxidase-conjugated anti-mouse IgG from Amersham Biosciences (Orsay, France). For immunofluorescence, anti-glial fibrillary acidic protein (GFAP) antibodies were purchased from Cell Signalling (Ozyme, St Quentin Yvelines, France), microtubule associated protein 2 (MAP2) from Abcam (Paris, France), macrosialin or murine homologue of the human CD68 from AbD Serotec (Düsseldorf, Germany), anti-P_T451_-PKR from Biosource (Nivelles, Belgium), secondary antibodies from DakoCytomation, (Trappes, France) and IgG- and protease-free bovine serum albumin (BSA) from Jackson ImmunoResearch Europe Ltd (Interchim distributor, Montluçon, France).

### Primary murine mixed neuron-astrocyte-microglia cultures

First, primary glial cultures were prepared from C57BL/6J mouse embryos of 18 days. Brains were quickly removed, and cerebral cortico-hippocampal regions were dissected in ice-cold and sterile 1X PBS (154 mM NaCl, 1.54 mM KH_2_PO_4_, 2.7 mM Na_2_HPO_4 _7H_2_O, pH 7.20 ± 0.05) containing 18 mM glucose and 1% PS as previously described [38]. Cells were then dissociated mechanically using a pipette into DMEM/1% PS, transferred into tubes containing FBS at the bottom (1 mL/30 mL cell suspension) and centrifuged at 300 × *g *for 10 min at 4°C. The cell pellet was suspended into DMEM/1% PS and centrifuged again. This step was repeated once. After the centrifugation, cells were suspended into DMEM/10% FBS/1% PS, seeded at a density of 4 × 10^5 ^cells/mL in Nunc EasYFlask™ (75 cm^2^) coated with 0.001% poly-L-lysine and then incubated at 37°C in a humidified 5% CO_2 _atmosphere. Medium was replaced every five days. These cells were cultured until day 14, the day of microglia purification.

Second, primary cultures with neurons and astrocytes were prepared from cortex and hippocampus of C57BL/6J mouse embryos of 18 days as above. Cells were suspended in MEM/Neurobasal (1:1) supplied with 18 mM glucose, B-27 Supplement, 1% glutamine, 2.5% FBS, 2.5% horse serum and 1% PS, and seeded in 6-well plates (10^6 ^cells per well) coated with 0.001% poly-L-lysine. Cultures were then maintained at 37°C in a humidified 5% CO_2 _atmosphere. At day 5, neurons and astrocytes were cultured with microglia purified from the primary culture described above.

Third, microglia were purified from glial cultures on day 14 as previously described with some modifications [39]. Briefly, confluent glial cultures were dissociated with trypsin/EDTA and cell suspensions were suspended in 1 mL of 70% isotonic Percoll and transferred into a 5 ml glass tube. Two mL of 50% isotonic Percoll were gently layered on top of the 70% layer and then 1 mL of 1X PBS layered on top of 50% isotonic Percoll layer. Tubes were centrifuged at 1200 × *g *for 45 min at room temperature (RT) with a program including minimum acceleration and brake in a swinging bucket rotor.

Purified microglia occupied the interface between 70 and 50% isotonic Percoll. The top interface between 1X PBS and 50% isotonic Percoll containing all other central nervous system (CNS) elements was carefully removed and microglia layer was transferred into a new tube and washed twice by adding 1 mL PBS and centrifuged at 500 × *g *for 5 min at RT. Cells were counted and seeded at the density of 150,000 cells per well into 6-well plates containing the primary culture of neurons/astrocytes to 5 days old in order to obtain a density of microglia close to that already described. Indeed, the density of microglia in the CNS of the normal adult mouse brain is variable depending on the brain region and represents 5% in the cerebral cortex, according to Lawson et al. [40]. The mixed murine co-cultures with neurons, astrocytes and microglia were then used three days later for experiments and a fourfold confocal staining with cell and nucleus markers (DAPI, MAP-2, GFAP, CD68 for nuclei, neurons, astrocytes and microglia, respectively) was investigated in cells seeded on poly-L-lysine-coated glass coverslips to quantify neurons, astrocytes and microglia. In additional file 1, figure S1, we show that neurons, astrocytes and microglia represent about 36, 57 and 6% of total cells, respectively, i.e. close to what is physiologically observed in the cortex.

### Chemical treatments

Co-cultures were treated with either C16 (specific inhibitor of PKR) at different concentrations (210 nM (IC50) and 1 μM) or DMSO (vehicle of C16) at less than 1%, in serum-free MEM:Neurobasal (1:1)/1% glutamine/1% PS medium 1 hour before 20 μM Aβ42 (or exactly 11 nmol in 550 μL of medium in each well receiving Aβ42) for 72 h at 37°C. Aβ42 was previously incubated 48 h at 37°C for aggregation as recommended by the Merck Chemical supplier [41]. The concentration of Aβ42 was chosen based on previous work in primary cultures [38,42]. After treatment, media were conserved in order to analyse Aβ42 monomers and oligomers by immunoblotting and fibrillar form of Aβ42 by scanning electron microscopy in our experimental conditions (see the additional file 2, Figure S2). Results show the presence of a mix composed with monomers, oligomers (8 and 12 kDa) and a dense network of fibrils. As the specific toxicity of these different states of Aβ is not clearly demonstrated, we decided to incubate cells with this whole mixture.

### Cell lysis and nuclear extracts

After treatment, media were stored at -80°C until used for ELISA of cytokines. Cells were then washed with PBS and lysed in ice-cold lysis buffer (50 mM Tris-HCl, 50 mM NaCl pH 6.8, 1% (v/v) Triton X-100, 1 mM PMSF, 50 mM NaF, 1% (v/v) protease inhibitor and 1% (v/v) phosphatase inhibitor cocktails). Lysates were sonicated for 10 sec and centrifuged at 15,000 × *g *for 15 min at 4°C. The supernatants were collected and analyzed for protein determination using a protein assay kit (Biorad, Marnes-la-Coquette, France). Samples were frozen at -80°C until further analysis.

Nuclear extracts were prepared as previously described [43]. Firstly, the cytoplasmic fraction was isolated and discarded, and the nuclear pellet was then lysed in nuclear lysis buffer (20 mM Hepes pH 7.9, 400 mM NaCl, 1 mM EDTA, 1 mM EGTA, 1 mM DTT, 0.5 mM PMSF, 1% of protease and 1% phosphatase inhibitor cocktails) during 2 h at 4°C. Then, vials were centrifuged at 1,600 × *g *for 5 min at 4°C and the supernatant was isolated. The quantity of total protein was measured with a Biorad protein assay kit.

### Enzyme-linked immunosorbent assay (ELISA)

Commercially available ELISA kits were used for assessing TNFα (sensitivity: 2 pg/mL), IL-1β (sensitivity: 15 pg/mL) and IL-6 (sensitivity: 2 pg/mL) according to the manufacturers' instructions (BioLegend, Ozyme, St Quentin Yvelines, France). The range of analysis was between 7.8-6000 pg/mL. Cell lysates were diluted (1:2) with the assay diluents and all steps were performed at RT. The enzymatic reaction was stopped after 15 min incubation with tetramethylbenzidine (TMB) substrate by adding 2N H_2_SO_4 _and the optical density (OD) was read at 450 nm within 30 min, using the Multiskan^® ^spectrum spectrophotometer. The cytokine levels were then calculated by plotting the OD of each sample against the standard curve. The intra- and inter-assay reproducibility was > 90%. OD values obtained for duplicates that differed from the mean by greater than 10% were not considered for further analysis. For convenience all results are expressed in pg/mL and in pg/mg protein for culture medium and cell lysates, respectively.

### Immunoblotting

Samples (30 μg proteins of cell lysates or nuclear extracts) were prepared for electrophoresis by adding NuPAGE^® ^LDS 4X LDS sample buffer and NuPAGE^® ^Sample Reducing Agent (10X). Samples were then heated up 100°C for 5 min and loaded into NuPAGE^® ^Novex^® ^Bis-Tris Mini Gels, and run at 200 V for 35 min in NuPAGE^® ^MES SDS running buffer containing 0.5% NuPAGE^® ^antioxidant. Gels were transferred to nitrocellulose membranes using the iBlot^® ^Dry blotting system set to program 20V for 7 min. Membranes were washed for 10 min in Tris-buffered saline/Tween (TBST: 20 mM Tris-HCl, 150 mM NaCl, pH 7.5, 0.05% Tween 20) and blocked 2 h in TBST containing 5% non fat milk or 5% bovine serum albumin (BSA).

Blots were incubated with primary antibody in blocking buffer overnight at 4°C. Antibodies used were rabbit anti-P_T451_-PKR (1:100), mouse anti-P_S32/36_-IκB (1:500), rabbit anti-IκB (1:500), rabbit anti-P_S536_-NF-κBp65 (1:500), rabbit anti-NF-κBp65 (1:500) and rabbit anti-caspase3 8G10 (1:500) which detects endogenous levels of full-length and large fragments of caspase-3 resulting from cleavage at aspartic acid 175. Membranes were washed 2 times with TBST and then incubated with the peroxidase-conjugated secondary antibody either anti-rabbit or anti-mouse IgG (1:1000) according to the origin of primary antibody during 1 hour at RT. Membranes were washed again and exposed to the chemiluminescence ECL luminol plus western blotting system (Amersham Biosciences, Orsay, France) followed by signal capture with the Gbox system (GeneSnap software, Syngene, Ozyme distributor). After 2 washes in TBST, membranes were probed with mouse antibody against tubulin (1:10000) or actin (1:100000) overnight at 4°C. They were then washed with TBST, incubated with peroxidase-conjugated secondary antibody anti-mouse (1:1000) for 1 h, exposed to the chemiluminescence ECL luminol western blotting system and signals were captured. Automatic image analysis software was supplied with Gene Tools (Syngene, Ozyme distributor). *Ratios *protein/tubulin or actin were calculated and are shown in the corresponding figures.

### Immunofluorescence

After treatment, cells on coverslips were washed once with PBS and fixed with 4% PFA for 15 min at RT. After three washes with PBS, the permeabilizing and blocking PBS buffer (137 mM NaCl, 2.7 mM KCl, 1.7 mM KH_2_PO_4_, 10.14 mM Na_2_HPO_4_, pH 7.4 containing 0.3% triton X-100 and 5% of IgG- and protease-free BSA) was added during 1 h at RT.

Staining of neurons, astrocytes and microglia was performed by incubating coverslips overnight at 4°C with a mix containing rabbit anti-MAP2 (1:50), mouse anti-GFAP (1:100) and rat anti-CD68 (1:25) in PBS containing 0.3% triton X-100 and 1% of BSA. Cells were then rinsed twice with PBS before 1 h incubation at RT with the mix containing secondary antibodies: swine anti-rabbit FITC (1:20), goat anti-mouse AlexaFluor 647 (1:25) and goat anti-rat R-Phycoerythrin (RPE) (1:25) diluted in PBS/0.3% triton X-100/1%BSA. Finally, cells were washed twice in PBS and twice in distilled water before using the Prolong Gold antifade reagent with DAPI.

Staining of P_T451_-PKR and cell marker (MAP2, GFAP or CD68) was performed in PBS/0.3% triton X-100/1% BSA overnight at 4°C by using rabbit anti-P_T451_-PKR (1:25) with chicken anti-MAP2 (1:100) and mouse anti-GFAP (1:100). After incubation, cells were washed twice with PBS before incubated with swine anti-rabbit (1:30) conjugated with tetramethylrhodamine isomer R (TRITC), goat anti-chicken FITC (1:50) and goat anti-mouse AlexaFluor 647 (1:25) for 1 h at RT. A sequential labelling for P_T451_-PKR and CD68 was performed. Firstly, cells were incubated with anti-CD68 antibodies overnight at 4°C, washed and incubated with goat anti-rat RPE. Secondly, cells were incubated with anti-P_T451_-PKR overnight at 4°C, washed and incubated with swine anti-rabbit FITC (1:20). Finally, coverslips were washed and mounted as described above.

Annexin V-FITC labels phosphatidylserine sites on the membrane surface. The kit used also includes propidium iodide (PI) to label cellular DNA in necrotic cells where the cell membrane has been totally compromised. For this labelling, cells were incubated with annexinV-FITC (1:50) and PI (1:100) in 1X binding buffer for 10 min at RT. Cells were then fixed with 4% PFA for 15 min at RT. After three washes with PBS, cells were incubated in the permeabilizing and blocking PBS buffer for 1 h at RT and with anti-MAP2 and anti-GFAP or with anti-CD68 in the same experimental conditions as described for the previous staining of P_T451_-PKR.

Multiply labelled samples were examined with a spectral confocal FV-1000 station installed on an inverted microscope IX-81 (Olympus, Tokyo, Japan) with Olympus UplanSapo x60 water, 1.2 NA, objective lens. Fluorescence signal collection, image construction, and scaling were performed using the control software (Fluoview FV-AS10, Olympus). Multiple fluorescence signals were acquired sequentially to avoid cross-talk between image channels. Fluorophores were excited with 405 nm line of a diode (for DAPI), 488 nm line of an argon laser (for Alexa 488 or FITC), 543 nm line of an HeNe laser (for TRITC and RPE) and the 633 nm line of an HeNe laser (for AlexaFluor 647). Emitted fluorescence was detected through spectral detection channels between 425-475 nm and 500-530 nm, for blue and green fluorescence, respectively and through a 560 nm and a 650 nm long pass filters for red and far red fluorescence, respectively. The images then were merged as an RGB image.

### Scanning electron microscopy

Cells were seeded on poly-L-lysine-coated glass coverslips at the same density described above. Treated primary co-cultures were rinsed briefly with PBS and fixed for 2 h at 4°C with 100 μM phosphate buffer (pH 7,4) containing 3% glutaraldehyde. After several rinses, they were post-fixed 1 h in 1% osmium tetroxide. Cells were washed again and dehydrated in acetone. Thereafter, samples were critical point-dried with a BAL-TEC CPD 030 using acetone and liquid carbon dioxide as the transition fluid. The dried specimens were coated with gold (25-35 nm thickness) using a sputtering device (BAL-TEC LCD 005). The samples were examined and photographed with a JEOL JSM-840 electron microscope.

### Statistics

Results are expressed as means ± SEM. Data for multiple variable comparisons were analysed by a one-way ANOVA followed by a Newman-Keuls' test as a post hoc test according to the statistical program GraphPad Instat (GraphPad Software, San Diego, CA, USA). The level of significance was p < 0.05.

## Results

### Toxicity of compound C16 in primary murine mixed co-cultures

Compound C16 is one of the most specific valuable imidazolo-oxindole inhibitors of PKR autophosphorylation that also rescues a PKR-induced translational block in a rabbit reticulocyte lysate system at micromolar concentrations [37]. Furthermore, previous data have shown that 1 μM C16 markedly reduces levels of P_T451_-PKR and caspase-3 activity in Aβ42-treated SH-SY5Y cells [27,43]. The T451 phosphorylated site in the PKR activation loop is required *in vitro *and *in vivo *for high-level kinase activity [44].

**Figure 1:**
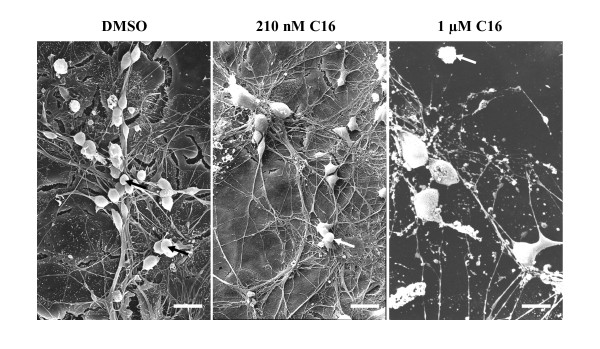
**Cytotoxicity of compound C16 in neuron/astrocyte/microglia co-cultures**. Scanning electron micrographs of neuron/astrocyte/microglia co-cultures prepared from brains of E18 C57Bl/6 mice. These co-cultures were incubated with the specific PKR inhibitor, C16 at 210 nM or 1 μM, or its DMSO vehicle in serum-free medium. The samples were examined in a JEOL JSM-840 electron microscope. Black or white arrows point out some microglia cells. Microglia appear as spherical smooth cells in contact with neurons and the astroglial layer in DMSO or 210 nM C16 conditions. However, in the presence of 1 μM C16, the integrity of cells is greatly altered with disruption of the axonal network, activated astrocytes with stellar form and microglia that look like macrophages. Scale bars: 10 μm, 15 μm and 20 μm for DMSO, 210 nM C16 and 1 μM C16, respectively.

We first evaluated toxicity of compound C16 at 210 nM (IC50) and 1 μM compared to its DMSO vehicle ( < 1%). By using scanning electron microscopy, we showed that the majority of cultured cells were neurons and astrocytic glial cells (Figure 1). Amongst these were some round cellular elements ranging from 10 to 15 μm in diameter which were identified as microglia cells. In experimental conditions with DMSO or 210 nM C16, microglia looked like spherical smooth cells in contact with neurons and the astroglial layer. No reactive microglia were observed in these control conditions. However, 1 μM C16 greatly affected the integrity of cells in co-cultures, with neuronal death, disruption of axonal network and activated astrocytes. The microglia looked like macrophages (Figure 1). Based on these observations, further experiments were performed with the effective concentration 210 nM, corresponding to IC50 of compound C16 [37].

### Prevention of Aβ-induced PKR activation and NF-κB/IκB signaling pathway by compound C16

**Figure 2:**
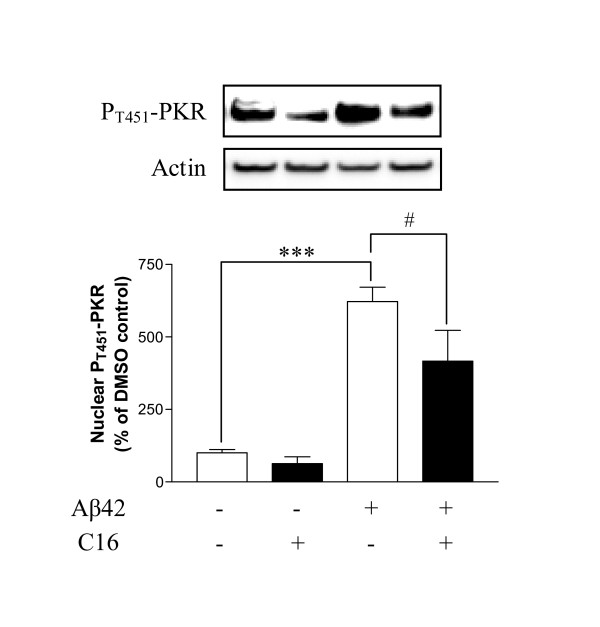
**Immunoreactivity of P_T451_-PKR in mixed co-cultures**. Representative blots showed immunoreactivity of P_T451_-PKR and actin in nuclear extracts of astrocyte/neuron/microlgia co-cultures treated with either 210 nM C16 or DMSO and exposed to 20 μM Aβ42 for 72 hours at 37°C. Wells of blots correspond to DMSO, C16, Aβ42 and DMSO, Aβ42 and C16, from left to right. Data are reported relative to actin. Results are expressed as percentage of DMSO control (set at 100%) and are mean ± SEM for 4 experiments. #p < 0.05, ***p < 0.001 compared to respective controls without C16 by one-way ANOVA with a Newman-Keuls multiple comparison test.

**Figure 3:**
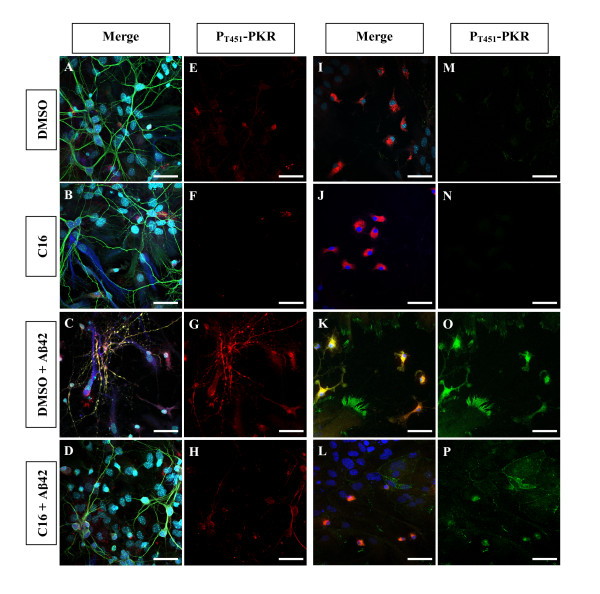
**P_T451_-PKR staining after C16 treatment and Aβ42 exposure in mixed co-cultures**. **(A to H) **Confocal staining of P_T451_-PKR (red channel), MAP2 (green channel), GFAP (blue channel) and DAPI in Aβ42-treated cells previously incubated with DMSO (C, G) or 210 nM C16 (D, H) compared DMSO (A, E)- or C16 (B, F)-treated cells. **(I to P) **Confocal staining of P_T451_-PKR (green channel), CD68 (red channel) and DAPI (blue channel) in co-cultures in the same experimental conditions described above. PKR was activated under Aβ42 exposure (G) compared to DMSO- and C16-treated cells (E and F, respectively). P_T451_-PKR is present in neurons, with an intense perinuclear, nuclear and axonal staining compared to DMSO-treated cells (Fig. 3: C, G compared to A, E). Neurons with nuclear P_T451_-PKR are shrunken (Fig. 3: C, G). Treatment with C16 decreased perinuclear and nuclear staining, but some axons remained stained (Fig.3: D, H). No signal of P_T451_-PKR was observed with only C16 (Fig.3: B, F). In astrocytes, a diffuse cytoplasmic staining of PT451-PKR and a robust staining in spine-like structures of astrocytic processes with Aβ42 (Fig.3: C, G) is observed and well prevented by C16 treatment (Fig.3: D, H). Microglia are activated with appearance of thick processes and irregular shape and display a high level of activated PKR after 72 h Aβ42 exposure (Fig.3: K, O) compared to DMSO-treated cells (Fig.3: I, M). C16 partially rescued this activation of PKR in microglia with no thick processes around cell bodies as in DMSO- or C16 conditions alone (Fig.3: L, P compared to I, M and J, N). Scale Bars: 42 μm.

At its IC50, C16 significantly reduced by 33% the prominent activation of PKR induced by 20 μM Aβ42 over 72 h in the co-cultures as shown by immunoblotting from nuclear extracts (Figure 2). Confocal staining of P_T451_-PKR confirmed the activation of PKR under Aβ42 exposure compared to DMSO-treated cells (Figure 3C, G and 3K, O*versus *3A, E and 3I, M, respectively). Moreover, co-staining with the neuronal marker MAP2 indicated that P_T451_-PKR was present in neurons, with intense perinuclear, nuclear and axonal staining, compared to DMSO-treated cells (Figure 3: C, G compared to A, E). Treatment with C16 decreased perinuclear and nuclear staining induced by Aβ42, but some axons remained stained (Figure 3D, H). The co-cultures incubated with compound C16 alone resembled those incubated with DMSO alone (Figure 3B, F, J, N*versus *3A, E, I, M, respectively). In astrocytes labeled by antibodies against GFAP, a diffuse cytoplasmic staining of P_T451_-PKR and a robust staining in spine-like structures of astrocytic processes with Aβ42 (Figure 3C and 3G) were observed and were well prevented by C16 treatment (Figure 3D, H). Microglia stained with anti-CD68 antibodies displayed a high level of activated PKR after 72 h of Aβ42 exposure (Figure 3K, O) compared to DMSO-treated cells (Figure 3I, M). There was also a change in cellular morphology; microglia were activated with appearance of thick processes and irregular shape with Aβ42 treatment (Figure 3K, O). C16 partially rescued this activation of PKR in microglia. Furthermore, we found only microglia with no thick processes around cell bodies as with C16 alone (Figure 3. merge images J and L).

**Figure 4:**
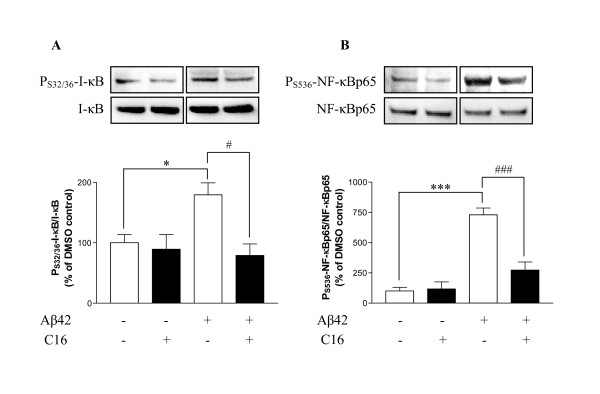
**Activation of IκB and NF-κB in lysates of murine astrocyte/neuron/microglia co-cultures**. Representative blots showed the immunoreactivity of P_S32/36_-IκB (A), P_S536_-NF-κB (B) and corresponding total proteins in lysates of murine astrocyte/neuron/microglia co-cultures pretreated with 210 nM C16 and exposed to 20 μM Aβ42 for 72 h. Wells of blots correspond to DMSO, C16, Aβ42 and DMSO, Aβ42 and C16 from left to right. Results of phosphorylated proteins are reported relative to total protein. Results are expressed as a percentage compared to DMSO-treated cells (set at 100%). Results are mean ± SEM derived from 5 experiments in duplicate. #p < 0.05, ###p < 0.001 and *p < 0.05, ***p < 0.001 compared to respective controls without C16 by one-way ANOVA with Newman-Keuls multiple comparison test.

The same experimental conditions were followed to study activation of the NF-κB/IκB signaling pathway. Results obtained by immunoblotting from cell lysates are presented as the *ratio *of phospho-protein/total protein in order to evaluate the activation of both proteins. The results show a significant increase in phosphorylation of IκB at serine 32-36 (79%) and NF-κB at serine 536 (629.8%) with Aβ42 exposure (Figure 4). p65-mediated transcription is regulated by S536 phosphorylation in the transactivation domain (TAD) by a variety of kinases (TRAF family member-associated NF-κB activator (TANK)-binding kinase (TBK), IKKα, and p38) through various signalling pathways. This phosphorylation enhances p65 transactivation potential [45-47].

Pre-incubation with 210 nM C16 significantly prevented activation of IκB and NF-κB compared to Aβ42-treated cells. The calculated *ratios *remained comparable to those obtained without Aβ42.

### Effects of compound C16 on Aβ-induced cytokine production and release in primary murine mixed co-cultures

**Figure 5:**
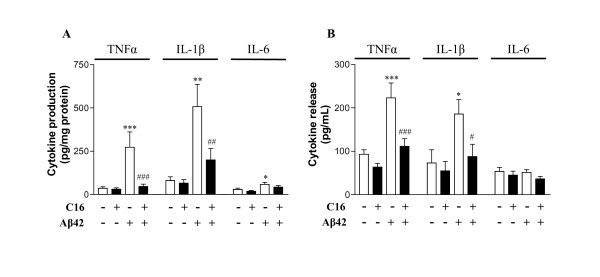
**Production (A) and release (B) of cytokines in murine astrocyte/neuron/microglia co-cultures**. These co-cultures were pretreated with 210 nM C16 or its DMSO vehicle and exposed or not to 20 μM Aβ42 for 72 h in serum-free medium. TNFα, IL-1β and IL-6 were assessed by ELISA. Data are expressed as mean ± SEM of pg/mg protein for production and pg/mL for release (n = 5 in duplicate). *p < 0.05, **p < 0.01, ***p < 0.001 compared to DMSO; #p < 0.05, ##p < 0.01 and ###p < 0.001 compared to Aβ42 DMSO after one-way ANOVA with Newman-Keuls multiple comparison test.

To determine the effect of PKR inhibition on cytokine levels in our cell lysates and released into the medium, samples were assayed by ELISA to quantify TNFα, IL-1β and IL-6 levels. Intracellular levels of these three cytokines were significantly higher in cells treated with 20 μM Aβ42 for 72 h (increase of 86.2% TNFα, 84% IL-1β and 50.6% IL-6) compared to DMSO-treated cells (Figure 5A). Treatment with 210 nM C16 significantly decreased levels of TNFα- and IL-1β-induced Aβ42 (83.2% and 60.7% inhibition, respectively) but failed to prevent IL-6 production (Figure 5A). Cytokine levels (TNFα and IL-1β) in Aβ42-exposed cells pretreated with 210 nM C16 were comparable to those measured in the absence of Aβ42 (Figure 5A).

Levels of released TNFα and IL-1β were also significantly increased after Aβ42 exposure (58.3% and 60.7% respectively) compared to DMSO-treated cells (Figure 5B). As for produced cytokines, the Aβ42-induced release of TNFα and IL-1β was significantly prevented by 210 nM C16 (50.1% and 52.7% inhibition, respectively). No significant change was observed for released IL-6, but levels of produced and released IL-6 remained very low in our experimental conditions (Figure 5B).

### Effects of compound C16 on altered cellular morphology induced by Aβ42 treatment

**Figure 6:**
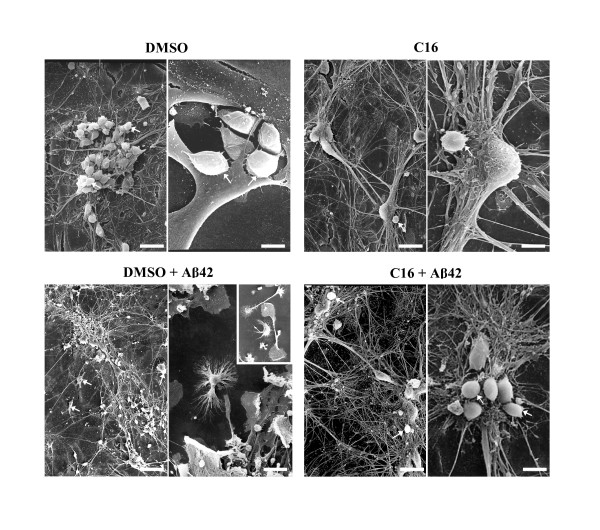
**Scanning electron micrographs of neuron/astrocyte/microglia co-cultures**. These co-cultures were preincubated with compound C16 at 210 nM or DMSO 1 h before treatment with 20 μM Aβ42 or distilled water for 72 h in a serum-free medium. As in figure 1, samples were examined in a JEOL JSM-840 electron microscope. Aβ42 strongly altered the axonal and dendritic network, compared to DMSO or 210 nM C16 conditions. Microglia are activated and display numerous spinous processes along cell bodies and cytoplasmic projections; some cells have undergone transformation into multipolar cells or cells with at least one thin process extending a distance greater than three times the cell body diameter, known as "process-bearing microglia". Some occasional short secondary branches were also observed. Insets showed different states of activated microglia in Aβ42-treated co-cultures. On the contrary, C16 prevented the activated state of microglia, which appear as smooth cells with few spines as in DMSO or C16 without Aβ42 treatment. While some neurons were dead compared to DMSO alone, the network of axons and dendrites is preserved and comparable to the network observed in DMSO or C16 conditions. Bars: 17 μm and 5 μm for DMSO and 15 μm and 6 μm for C16, 35 μm and 10 μm for DMSO + Aβ42, 14 μm and 6 μm for C16 + Aβ42, at low and high magnification, respectively. White arrows indicate microglia cells.

As we have shown before, Aβ42 induced the NF-κB signaling pathway and cytokine production, which were prevented by the inhibitor of PKR, compound C16. The beneficial effect of C16 has also been analyzed by using scanning electron microscopy. In micrographs, 20 μM Aβ42 largely affected co-cultures, producing massive neuronal loss (Figure 6). Axonal and dendritic networks were also altered with many disruptions of axons and dendrites, which clearly appeared thinner than with DMSO or 210 nM C16 treatments. Microglia were activated and different morphological changes were observed: microglia cells displayed numerous spiny processes along their cell bodies and cytoplasmic projections, and some cells underwent transformations into multipolar cells or cells with at least one thin process extending a distance greater than three times the cell's body diameter, known as "process-bearing microglia". Some occasional short secondary branches were also observed (see inset in micrograph of Aβ42-treated co-culture in Figure 6). On the contrary, in C16/Aβ42 experimental conditions, microglia looked like smooth cells with few spines as with DMSO or C16 treatment without Aβ42 treatment. While some neurons were dead, compared to treatment with DMSO alone, the network of axons and dendrites was preserved and comparable to the network observed with DMSO or C16 treatments (Figure 6).

### Effects of compound C16 on Aβ42-induced apoptosis

**Figure 7:**
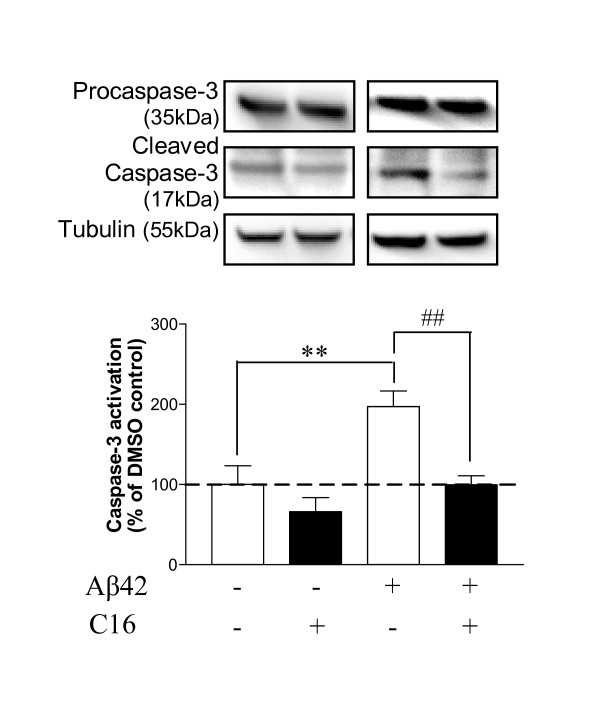
**C16 prevented activation of caspase-3**. Representative blots show the immunoreactivity of procaspase-3, cleaved caspase-3 fragments and tubulin in astrocyte/neuron/microglia co-cultures pretreated with 210 nM of C16 compared to vehicle treatment and with or without 20 μM Aβ42 exposure for 72 h. Data of cleaved-caspase-3 are reported relative to data of corresponding procaspase-3. Results are expressed as percentage of DMSO condition (set at 100%) and are mean ± SEM for 4 experiments in duplicate. ##p < 0.01 and ***p < 0.001, compared to respective controls without C16 by one-way ANOVA with Newman-Keuls multiple comparison test.

Caspase-3 is known to be a crucial mediator of apoptosis through its protease activity. Activation of caspase-3 requires proteolytic processing of its inactive zymogen into activated fragments after cleavage at aspartic acid 175. In order to evaluate apoptosis in cell co-cultures, we studied the activation of caspase-3 in cell lysates represented by the *ratio *of cleaved-caspase-3/total caspase-3 (Figure 7). Results show a great increase in activation of caspase-3 after Aβ42 exposure for 72 h (97%) compared to DMSO-treated cells. This activation was totally prevented by 210 nM C16, and the *ratios *were comparable to those obtained in DMSO-treated cells.

**Figure 8:**
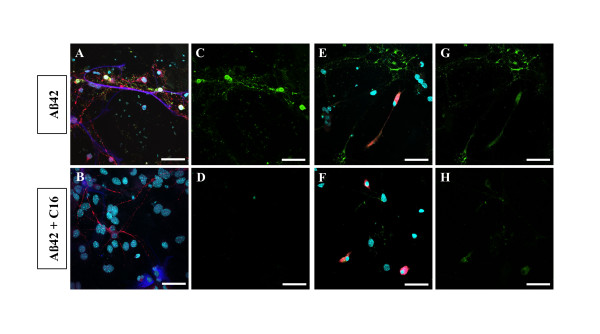
**C16 prevented apoptosis in neurons**. Confocal staining of annexin V-FITC (green channel) with MAP2 (red channel) and GFAP (blue channel) (A, B, C and D) or with CD68 (red channel) (E, F, G and H) after 20 μM Aβ42 exposure and treated with 210 nM of C16 or DMSO for 72 h. A, B, E and F are merged images and C, D, G and H show only annexin-V-FITC staining. DAPI was used as a nuclear stain. A strong annexin V-FITC staining colocalizes with MAP2 staining after Aβ42 exposure, whereas GFAP-positive cells appear unaffected (A, C). We found also a diffuse and very weak staining of annexin V-FITC in CD68-positive cells (E, G). C16 prevented translocation of phosphatidylserine from the inner to the outer plasma membrane leaflet both in neurons (B, D) and microglia (F, H). Photos are representative of 4 experiments. Scale Bars: 40 μm.

During apoptosis, phosphatidylserine is translocated from the inner to the outer plasma membrane leaflet. This externalization was analyzed with annexin V-FITC staining to examine apoptotic states of the different cell populations after treatment with 210 nM C16 and Aβ42 exposure for 72 h (Figure 8). Furthermore, the apoptosis detection kit includes propidium iodide (PI) to label the cellular DNA in necrotic cells. This combination allows the differentiation among early apoptotic cells (annexin V-FITC-positive, PI-negative), necrotic cells (annexin V-FITC-positive, PI-positive), and viable cells (annexin V-FITC-negative, PI-negative). In all conditions examined, no PI staining associated with annexin V-FITC staining was observed. The state of necrotic cells was probably at a maximum, with complete nucleus destruction, explaining the lack of PI staining. Thus, co-staining annexin V-FITC and cell markers excluded PI incubation in our protocol. The results show that prominent annexin V-FITC staining colocalizes with MAP2 staining after Aβ42 exposure, whereas GFAP-positive cells appeared unaffected (Figure 8A and 8C). We found also a diffuse and very weak staining of annexin V-FITC in CD68-positive cells (Figure 8E and 8G). Exposure to 210 nM C16 yielded no evidence of apoptosis either in neurons (Figure 8B and 8D) or in microglia (Figure 8F and 8H).

## Discussion

Our previous findings indicated that PKR is associated with apoptotis in brains of APP_SL_PS1 knock-in transgenic mice, and *in vitro *in Aβ42-treated SH-SY5Y neuroblastoma cells [27,29,38,43]. Moreover, other studies have clearly reported that PKR is involved in the activation of NF-κB pathway through phosphorylation of IKK [32,48,49] and I-κB [33] in models of viral infection. NF-κB plays a critical role in many cellular events, such as expression of cytokine genes that affect inflammatory process. Concerning AD, NF-κB has been shown to be upregulated and responsible for the induction of TNFα, IL-1β and IL-6 mRNA [7,50-52], particularly in glial cells. Furthermore, many studies have shown that Aβ neurotoxicity induces cytokine production and release of TNFα, IL-1β and IL-6 [8,53,54]. This inflammatory process has also largely been described in brain [55,56] and in the periphery in plasma, serum or mononuclear cells of patients with AD [19,57]. Although inflammation might have a neuroprotective role through Aβ phagocytosis, it is of interest to better understand the regulation involved in production of inflammatory factors in AD in order to limit neuronal death when the inflammatory process switches to an unregulated phenomenon. Because of the involvement of PKR in NF-κB-mediated inflammation, we were interested in studying the effect of PKR inhibition on production of inflammatory factors in a murine mixed co-culture.

The cell culture model used in this project is an embryonic (E18) mouse brain co-culture that includes neurons (36%), astrocytes (57%) and microglia (6%), in order to reflect the cell population in normal adult mouse cortex [40]. In control conditions without amyloid stress, no inflammatory reactive glia were observed, excluding any trauma during cell preparation. The major aim with this model was to be close to physiologic conditions and to recreate *in vitro *the essential neuron/glia environment to explore the effects of the inflammatory process on neurons. Currently, independent cultures of microglia or astrocytes with or without neurons are widely used as models of inflammation in brain. However, it seemed essential to maintain these three cellular actors together in our experimental conditions, considering the multiple interactions between neurons and glia, in particular in inflammatory conditions [58-61]. This model is produced from embryonic tissue, and one must therefore remain cautious about its use because, as we know, the maturity of the regulatory and compensation processes is not complete. The cells may be more or less vulnerable to the toxicity of amyloid peptide compared with adult cells. Their tolerance system has not yet been sufficiently explored. In addition, the concentration of exogenous amyloid peptide added in cultures, although identical to that used in many published studies, is far greater to that found in brains of patients with Alzheimer's disease. However, it is known that levels of both Aβx-40 and Aβx-42 increase very early in the disease process, and in the frontal cortex these increases occurr in the absence of significant neurofibrillary pathology. These levels increase systematically with severity of cognitive decline contrary to Aβ burden as assessed only in neuritic plaques [62].

For this mixed co-culture model, we have shown that the PKR inhibitor at a concentration of 1 μM, as previously used on neuroblastoma cell line [43], induces a great alteration, leading us to use a lower concentration of 210 nM corresponding to the IC50 [37]. This concentration was effective in inducing a decrease of PKR phosphorylation on threonine 451 by 33% in cells exposed to 20 μM Aβ42 for 72 h.

By immunostaining, we showed that Aβ42 induces activation of PKR in neurons with a perinuclear and nuclear localization as we have previously described [38], but also in glia where PKR is highly activated in spine-like structures of astrocytic processes and in the cytoplasm of microglia. Expression of PKR is known in astrocytes to be among an array of receptors involved in innate immunity [63] but this expression has not yet been described in microglia. Treatment of these three cellular types with 210 nM C16 before Aβ42 exposure for 72 h decreased P_T451_-PKR staining, but a residual amount of activated PKR remained. These findings were also associated with a more preserved integrity of the cells compared to Aβ42-treated cultures without C16. Indeed, two spectacular cellular events were clearly protected: the dendritic and axonal network of neurons and the morphology of microglia. In Aβ conditions, many of the neurons showed signs of neuritic damage with beading and fragmentation, according to other studies [64-66], and formation of pleiomorphic microglia was observed with ramified microglia and features of chronically activated microglial cells represented by a markedly elongated cells named rod microglia. In brains of patients with AD, activated rod and ramified microglia are observed: ramified microglia are in contact with amyloid fibrils and rod microglia are found predominantly at the edge of senile plaques [67,68]. For astrocytes, morphological modifications were very limited with thinner extensions. This mixed co-culture model of AD displayed the morphological degeneration and glial activation seen in AD, which was rescued by pretreatment with C16.

Besides the role of C16 in the rescue of the integrity of co-cultures, we found that this PKR inhibitor induced also a significant decrease in Aβ42-induced I-κB and NF-κB activation, bringing their activation rates back close to those observed without exposure. A previous study using the overexpression of sirtuin 1 (SIRT1) deacetylase and the addition of the SIRT1 agonist resveratrol showed markedly reduced NF-κB signaling stimulated by Aβ with strong neuroprotective effects in primary mixed neuronal/glial cultures from rat cortices [69]. Moreover, it is interesting to note that inhibition of the many kinases involved in the NF-κB pathway by META060 showed an ability to suppress *in vitro *and *ex vivo *LPS-mediated inflammation [70].

Taken together, these results led us to investigate cytokine production and release after C16 treatment. Our results obtained by ELISA show a robust inhibition of Aβ42-induced production and release of both TNFα and IL-1β but, surprisingly, we did not find any modification for IL-6 by pretreatment with C16. While levels of IL-6 were significantly higher than in vehicle conditions, the amounts remained very low whatever the conditions. It is known that astrocytes are the major source of IL-6 in CNS injury and inflammation [71]. Many stimuli can upregulate IL-6 production, in particular TNFα and IL-1β [72], but concentrations required to induce IL-6 production in human astrocytes are higher than 1 ng/mL [73] whereas, in our model, concentrations of TNFα and IL-1β were lower than 600 pg/mL. Although we showed a robust increase in TNFα and IL-1β after 72 h of Aβ42 exposure, it seems that this increase was insufficient to induce IL-6 production in astrocytes. Microglia can also produce IL-6, but a recent study revealed that microglia from young mice are less responsive to stimulation and secrete lower levels of IL-6 than do microglia from aged mice [74]. In addition, many studies have reported no modification of IL-6 expression or secretion in spite of IL-1β treatment of primary astrocytes or primary microglia cultures, with or without neurons [75] related to serum-free conditions [54], β-amyloid protein structure [76], or glutathione concentration [77]. While there are few experiments using mixed cultures of neurons and glial cells, one study showed that 10 μM Aβ42, previously aggregated, induced a decrease of IL-6 levels after two days of incubation [78]. These contradictory results regarding effects on IL-6 levels of Aβ *in vitro *have also been obtained for brains, peripheral cells, serum and plasma of patients with AD [8].

TNFα seems to be a critical mediator of the effects of neuroinflammation on early (pre-plaque) pathology in 3xTgAD mice, and its inhibition in the CNS may slow the appearance of amyloid-associated pathology, cognitive deficits, and potentially the progressive loss of neurons in AD [79]. These results support the observations made a year before concerning the inhibition of TNFα by thalidomide showing a capacity to prevent amyloid beta-induced impairment of recognition memory in mice treated by intracerebral ventricular injection of Aβ25-35 [80]. Finally, neutralizing the TNFα pathway by etanercept prevents behavioural changes in an inflammatory rat model obtained by microinjection of IL-1β into the hypothalamus [81].

It has also been shown that ibuprofen suppresses IL-1β induction and ameliorates β-amyloid pathology in APPswe (Tg2576) mice [82]. Thus, preventing both TNFα and IL-1β production would seem to be an efficient strategy to slow damage observed in AD models.

To check these literature data suggesting a protective effect of the regulation of inflammation, we studied the apoptotic state of our co-cultures. We show that beyond the inhibition of both Aβ42-induced TNFα and IL-1β production and release, cells in co-cultures display significant reduction of activated pro-apoptotic caspase-3 after PKR inhibitor treatment. Caspase-3 is able to cleave PKR to generate active PKR N-terminal and C-terminal fragments that play a role in the activation of intact PKR [42,83] and contribute to the apoptotic process [84]. Moreover, staining with annexin V-FITC has specified that apoptosis is induced in neurons with axonal processes drastically altered by Aβ42, according to previous studies [64], and that the PKR inhibitor completely prevents this initiation of apoptosis in neurons, displaying a preserved integrity. Although no positive PI staining associated with annexin V-FITC was observed, probably due to nuclear lysis, cellular debris are absent in the presence of compound C16, indicating also that this PKR inhibitor prevents Aβ42-induced necrosis. A signal of annexin V-FITC was also observed in a few activated microglia in Aβ42-treated co-cultures and we can underline that pretreatment with C16 rescued the morphology of microglia from rod microglia to round microglia and astrocytes from spider-like to protoplasmic structures. It is well known that caspase-3 is a key factor in TNFα- and IL-1β-induced apoptosis and neuronal loss in AD [85]. Moreover studies described a major role for TNFα and IL-1β in caspase-3 activation [86,87]. These findings are consistent with the prevention of apoptosis observed in our model through decreases of only TNFα and IL-1β.

In astrocytes and microglia, PKR, highly cytoplasmic, could be involved in the modulation of the production of inflammatory factors. This suggestion is supported by a study reporting PKR functions as an essential modulator in inflammatory signaling events. They revealed that activation of PKR by LPS leads to induction of interferon-β through activation of NF-κB, triggering phosphorylation of STAT1 in rat brain glial cells [88]. Furthermore, it was described that β-amyloid peptides induce degeneration of cultured rat microglia [89]. Thus, microglia might be unable to function normally and to properly respond to amyloid stimulus. Recent papers have underlined the senescence of microglia in AD, with loss of their neuroprotective properties, preceding the onset of tau pathology [90], suggesting that breakdown of the brain's immune system may be an important factor in the development of neurodegeneration [91]. PKR inhibition, which prevents Aβ42-induced morphologic alterations of microglia, could limit the degeneration of microglia and restore a normal profile of inflammatory functions.

## Conclusions

Our results highlight the involvement of PKR in the inflammatory response to Aβ42 by using primary murine mixed co-cultures allowing interactions between neurons, astrocytes and microglia. Interestingly, the significant decrease of Aβ42-induced cytokine production and release by a specific inhibitor of PKR was associated with preserved integrity of cells and rescue from apoptosis. Note that the compound C16 was added once before a 72 h-time incubation of mixed co-cultures with Aβ42, indicating its efficiency at IC50 in time. These findings could strengthen therapeutic strategies aimed at preventing deregulated inflammatory process in AD models through a very specific signaling pathway. In our laboratory, *in vivo *experiments with APPswePS1dE9 transgenic mouse model have been performed to determine if this specific PKR inhibitor could be relevant in the treatment of AD (data submitted).

## List of abbreviations

Aβ: β-amyloid; AD, Alzheimer's disease; APP: amyloid precursor protein; BSA: bovine serum albumin; CNS: central nervous system; COX2: cyclooxygenase 2; DAMPs: danger-associated molecular patterns; DAPI: 4',6-diamidino-2-phenylindole; DMSO: dimethylsulfoxide; DTT: dithiothreitol; FITC: fluorescein isothiocyanate; GFAP: glial fibrillary acidic protein; GSK-3β: glycogen synthase kinase 3β; IKK: IκB kinase; IL: interleukin; JNK: Jun kinase; LPS: lipolysaccharide; MAP2: microtubule associated protein 2; NaF: Sodium fluoride; NF-κB: nuclear factor-kappa B; NSAIDS: non-steroidal anti-inflammatory drugs; OD: optical density; PBMCs: peripheral blood mononuclear cells; PHFs: paired-helical filaments; PKR: double-stranded RNA-dependent protein kinase; PMSF: phenylmethylsulfonyl fluoride; PRRs: pattern recognition receptors; p38: MAP kinase 38 kDa; RAGE: receptor for advanced glycation end products; RPE: R-Phycoerythrin; RT: room temperature; SIRT1: sirtuin 1; TBST: tris-buffered saline Tween 20; TLRs: toll-like receptors; TMB: tetramethylbenzidine; TNFα: tumor necrosis factor; TRITC: tetramethylrhodamine isomer R.

## Competing interests

The authors declare that they have no competing interests.

## Authors' contributions

JC and GP designed the study and carried out all methods excepted the scanning electron microscopy performed by SM. RP participated in ELISA. MM participated in the preparation of mixed co-culture. MP participated in the conception of the study and has been involved in revising the manuscript critically for important intellectual content with FT and BF. All authors read and approved the final manuscript.

## Supplementary Material

Additional file 1 **Immunostaining of neurons, astrocytes and microglia in primary mixed murine cultures**. A fourfold confocal staining (DAPI, MAP-2, GFAP, CD68 for nuclei, neurons, astrocytes and microglia, respectively) was used to count neurons, astrocytes and microglia in mixed co-cultures. Click here for file

Additional file 2 **State of exogenous Aβ42 assembly in primary mixed murine cultures**. State of Aβ42 aggregations after 72 h incubation in neuron/astrocyte/microglia cultures using immunoblotting and scanning electron microscopy in our experimental conditions. Click here for file
