# Supplementary material for: Prevention of the β-amyloid peptide-induced inflammatory process by inhibition of double-stranded RNA-dependent protein kinase in primary murine mixed co-cultures
Source: J Neuroinflammation. 2011 Jun 23;8:72. doi: 10.1186/1742-2094-8-72 (PMC3131234; doi:10.1186/1742-2094-8-72)
Supplement: Additional file 1 — Immunostaining of neurons, astrocytes and microglia in primary mixed murine cultures. A fourfold confocal staining (DAPI, MAP-2, GFAP, CD68 for nuclei, neurons, astrocytes and microglia, respectively) was used to count neurons, astrocytes and microglia in mixed co-cultures. [file 1742-2094-8-72-S1.PDF]

Additional file 1

Figure S1. Immunostaining of neurons, astrocytes and microglia in primary mixed murine cultures

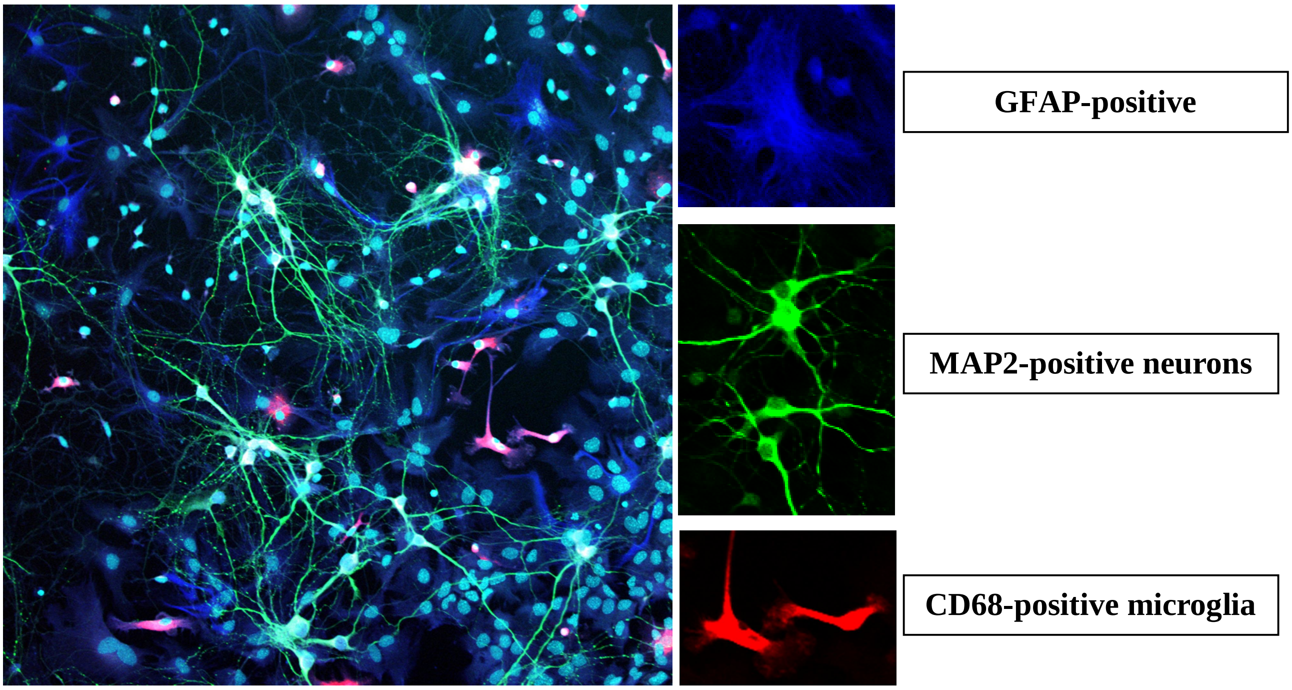

	Nuclei	Neurons	Astrocytes	Microglia
**mean ± SEM**	521 ± 36	179 ± 14	311 ± 24	30 ± 3
**Percentage of total cells ± SEM**	100	36.36 ± 1.87	57.44 ± 1.98	6.19 ± 0.60

Immunofluorescence was performed to identify astrocytes, neurons and microglia by using specific cell markers: GFAP, MAP2 and CD68, respectively. Total number of cells was determined by counting the nuclei dyed with DAPI. In five independent experiments, two fields per glass coverslips were counted (mean number of total cells/field: 521 ± 36) by using NIS-Elements software (Nikon, Badhoevedorp, The Netherlands). A *ratio* of the number of GFAP- or MAP2- or CD68-positive cells/DAPI-positive nuclei was calculated and expressed in percentage. Total number of cells was fixed to 100%. Scale bar for merge image: 37.5 μm. Higher magnifications for each cell type were showed on the right of merge image.
