# Supplementary material for: Prevention of the β-amyloid peptide-induced inflammatory process by inhibition of double-stranded RNA-dependent protein kinase in primary murine mixed co-cultures
Source: J Neuroinflammation. 2011 Jun 23;8:72. doi: 10.1186/1742-2094-8-72 (PMC3131234; doi:10.1186/1742-2094-8-72)
Supplement: Additional file 2 — State of exogenous Aβ42 assembly in primary mixed murine cultures. State of Aβ42 aggregations after 72 h incubation in neuron/astrocyte/microglia cultures using immunoblotting and scanning electron microscopy in our experimental conditions. [file 1742-2094-8-72-S2.PDF]

Additional file 2

Figure S2. State of exogenous Aβ42 assembly in primary mixed murine cultures.

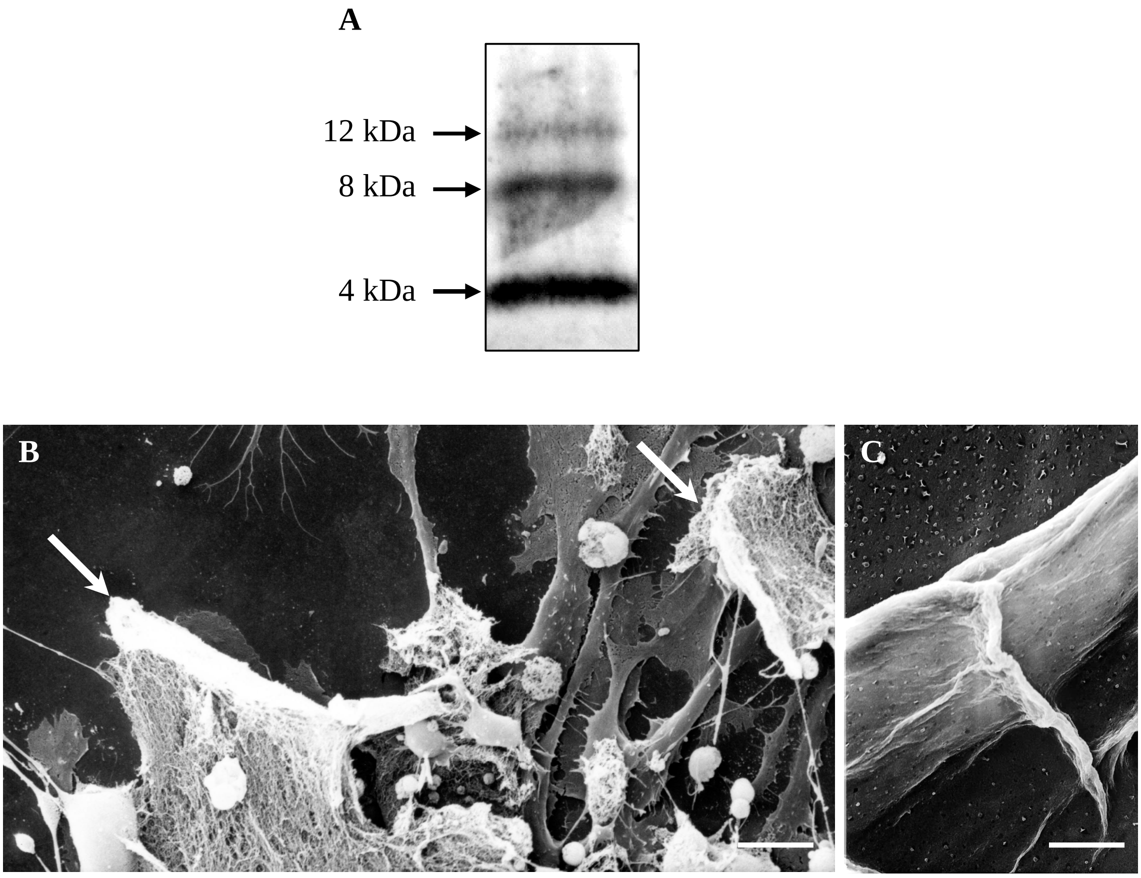

(A) Representative immunoblot showed Aβ42 previously incubated 48h for its polymerization and then after 72h at 20 µM (or exactly 11 nmol in 550 µL of medium in each well receiving Aβ42) in the culture medium at 37°C. The anti-amyloid antibody recognizes a specific band at 4 kDa for monomeric form and oligomers at 8 and 12 kDa under non-denaturing and non-reducing conditions. This anti-amyloid antibody (clone WO2) specifically recognizes between amino acid residues 4-10 of Aβ.

(B) Aβ42 fibrils were visualized by scanning electron microscopy in cells incubated with 20 µM Aβ42 in the same experimental conditions as above. White arrows indicated amyloid deposits in the co-culture. **(C)** Higher magnification of dense Aβ42 fibrils.

Scale bars: 7 µm (B) and 13 µm (C).
